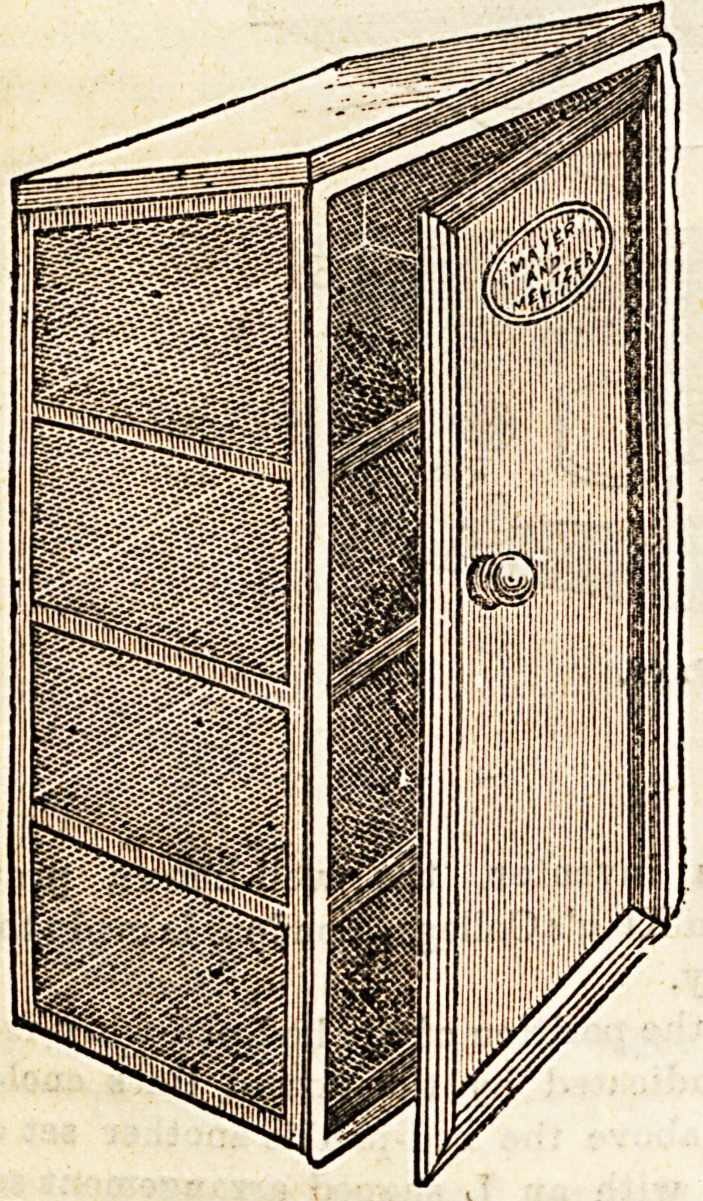# Excreta or Bed-Pan Cage

**Published:** 1892-05-14

**Authors:** 


### EXCRETA OR BED-PAN CAGE.

It would be difficult to speak too highly in praise of this arrangement, which we maintain should be found in every institution, whether a regular hospital or not. These cages, which are for storing the excreta of patients for the inspection of medical men, are perforated on three sides, and are fixed in an outer wall, the doors only being visible within the building, and these are made air-tight. From a sanitary point of view the cage is beyond all praise, and it also serves as a preventive of mistaken removal of excreta, which not infrequently happens, whereby serious delay is caused in diagnosis. With the use of the cage the employment of disinfectants can be omitted, an added advantage for purposes of analysis.

## Figures and Tables

**Figure f1:**